# Tumor Necrosis Factor-Like Weak Inducer of Apoptosis Activates Type I Interferon Signals in Lupus Nephritis

**DOI:** 10.1155/2017/4927376

**Published:** 2017-11-26

**Authors:** Leixi Xue, Lei Liu, Jun Huang, Jian Wen, Ru Yang, Lin Bo, Mei Tang, Yi Zhang, Zhichun Liu

**Affiliations:** ^1^Department of Rheumatology and Immunology, The Second Affiliated Hospital of Soochow University, Suzhou, China; ^2^Department of Rheumatology and Immunology, The First People's Hospital of Kunshan City, Kunshan, China

## Abstract

Type I interferon (IFN) plays a central role in pathogenesis of systemic lupus erythematosus (SLE); tumor necrosis factor-like weak inducer of apoptosis (TWEAK) has been associated with a pathogenic role in lupus nephritis (LN). Thus we investigated whether TWEAK could induce the activation of type I IFN pathway in LN. We examined this in patient-derived peripheral blood mononuclear cells (PBMCs) as well as MRL/lpr mice, a murine LN model. Relative to the control cohorts, MRL/lpr mice showed severe histological changes, high index levels of renal damage, and elevated expression of type I IFN-inducible genes. After shRNA suppression of TWEAK, we observed that renal damage was significantly attenuated and expression of type I IFN-inducible genes was reduced in MRL/lpr mice. In parallel, siRNA of TWEAK also significantly reduced the expression of type I IFN-inducible genes in PBMCs relative to control transfections. In PBMCs, TWEAK stimulation also led to expression of type I IFN-inducible genes. Our results illustrate a novel regulatory role of TWEAK, in which its activity positively regulates type I IFN pathway in LN based on preclinical models. Our findings suggest TWEAK could act as a critical target in preventing renal damage in patients with LN.

## 1. Introduction

Systemic lupus erythematosus (SLE) is a prototype autoimmune disease and affects the skin and hematologic, musculoskeletal, and renal organ systems [1]. Although genetic predisposition and environmental and hormonal factors are thought to be important to disease pathogenesis, the physiological and biochemical factors that promote SLE still remain unknown [2].

Interferon *α* (IFN*α*) is prototype cytokine of type I IFN, a family of proteins produced by most cells of the organism in response to a wide array of antigens such as apoptotic debris and viral particles [3]. In recent years, studies have indicated that type I IFN may play a critical role in SLE [4–9]. IFN gene signature has been used to classify SLE patients, and patients with high IFN gene signature are correlated with poor prognosis as measured using the SLE Disease Activity Index 2000 [10]. A modified IFN score, a sum of three type I IFN-inducible gene expression levels (lymphocyte antigen 6 complex locus E (LY6E), 2′,5′-oligoadenylate synthetase-like (OASL), and IFN*α*-inducible protein (clone IFI-15K) (ISG15)) or the LY6E level alone has also served as reliable biomarkers in diagnosing SLE [4].

Tumor necrosis factor-like weak inducer of apoptosis (TWEAK), a member of the TNF ligand superfamily, is expressed by many immunocytes and organs, including monocytes, dendritic cells, T cells, small intestine, heart, pancreas, colon, and kidney [11]. TWEAK was first described as an inducer of apoptosis in transformed cell lines [12]. More recently, it has been associated with a pathogenic role in lupus nephritis (LN) [13–16]. In nephrotoxic serum nephritis, a model of antibody-induced nephritis, anti-TWEAK mAb administration significantly ameliorated proteinuria and improved kidney histology, including a significant decrease in glomerular immunoglobulin deposition, infiltrated macrophage, and tubulointerstitial fibrosis [17]. TWEAK also promotes human kidney cells to locally express multiple inflammatory mediators [18, 19] and upregulates the levels of prostaglandin E2, matrix metalloproteinase 1, interleukin-8 (IL-8), and IL-6 in fibroblasts and synoviocytes [20].

In a human colorectal cancer cell line, TWEAK can signal through the JAK-STAT pathway to induce type I IFN, facilitating tumor cell apoptosis [21]. However, whether TWEAK mechanistically regulates IFN*α* expression in LN remains unclear. Therefore, this study aimed to specifically test a direct role of TWEAK in activating the type I IFN pathway. Our experiments leverage LN patient-derived peripheral blood mononuclear cells (PBMCs) as well as renal tissue from a mouse model of LN.

## 2. Materials and Methods

### 2.1. Patients Enrollment

The study protocol and all experimental procedures were approved by the Human Ethics Review Committee of the Second Affiliated Hospital of Soochow University, Suzhou, China. Twelve female patients with LN, 14 to 48 years that were treated in our department (Department of Rheumatology and Immunology, The Second Affiliated Hospital of Soochow University) between 2013 and 2014, were included in this study, according to the SLE and LN classification criteria of American College of Rheumatology (Supplementary Table 1) [22]. None of the patients received immunomodulatory therapy during the last month prior to blood collection.

### 2.2. Isolation and Culture of Peripheral Blood Mononuclear Cells

Isolation of peripheral blood mononuclear cells (PBMCs) was carried out using Ficoll (GE Healthcare, Uppsala, Sweden), according to the manufacturer's instructions. Purified human PBMCs were cultured in phenol red-free RPMI (Life Technologies, Grand Island, NY, USA) tissue culture medium containing 5% charcoal-stripped fetal bovine serum (Life Technologies, Grand Island, NY, USA). Afterward, they were divided equally into four groups and treated for 24 hours with one of the following: 5 × 10^−5^ mmol/L TWEAK-siRNA- (RIBOBIO, China) lipofectamine 2000 (Invitrogen, USA), 5 × 10^−5^ mmol/L control-siRNA- (RIBOBIO, China) lipofectamine 2000, 100 ng/mL recombinant human TWEAK (rhTWEAK, R&D Systems, Minneapolis, MN, USA), and normal saline (as controls).

### 2.3. Mice

MRL/lpr mice and MRL/MPJ mice (Shanghai Slack Laboratory Animal Co., Ltd., Shanghai, China) were bred and maintained in the animal facility of the Second Affiliated Hospital of Soochow University. 13-week-old MRL/lpr mice were administered through the tail vein with one of the following: 2 × 10^7^ transducing units (TU) lentivirus- (LV-) TWEAK-short hairpin RNA (TWEAK-shRNA group), 2 × 10^7^ TU LV-control-shRNA (control-shRNA group), and normal saline (as controls). The mice were sacrificed at day 29 after the treatment.

The shRNAs targeting mouse TWEAK gene (GenBank accession number: NM_011614) were designed using siRNA Target Finder and Design Tool (available at http://www.ambion.com) and were commercially obtained from GeneChem (Shanghai, China). The sequences of LV-TWEAK-shRNA and LV-control-shRNA are presented in Supplementary Table 2.

### 2.4. Quantitative Real-Time PCR

Total RNA was extracted from PBMCs or kidney tissue using Trizol (Invitrogen, Carlsbad, CA, USA) according to the manufacturer's instructions. Reverse transcription was performed with 3 *μ*g of total RNA using M-MuLV First Strand cDNA Synthesis Kit from Sangon Biotech (Shanghai, China). The sequences of primers of LY6E, OASL, ISG15, TWEAK, and *β*-actin were presented in Supplementary Table 3. Quantitative real-time PCR was performed in triplicate, using the SYBR green stain and the CFX96 Real-Time PCR Detection System (Bio-Rad Laboratories, Hercules, CA, USA), under the following conditions: 10 min at 95°C and 45 cycles of 95°C for 10 sec, 60°C for 20 sec, and 72°C for 30 sec. The cycle threshold (Ct) is the cycle at which the quantitative real-time PCR product crosses the detection threshold. The difference between target gene mRNA Ct value and *β*-actin mRNA Ct value is associated as ΔCt = Ct (target gene mRNA) − Ct (*β*-actin mRNA). The relative target gene mRNA abundance (ΔΔCt) represents the difference between the ΔCt values for a pair of conditions. The relative mRNA expression, assuming 100% quantitative real-time PCR efficiency, is exponential and is defined by the formula: [mRNA] = 2^−ΔΔCt^.

### 2.5. Western Blotting

Total cell lysates from kidney were collected in PBS and resuspended in a modified radioimmunoprecipitation assay (RIPA) lysis buffer (50 mM Tris-HCl, pH 8.0, 250 mM NaCl, 1% Nonidet P-40, 0.5% sodium deoxycholate, and 0.1% SDS), supplemented with protease inhibitor (Roche Diagnostics, Mannheim, Germany) and incubated at 4°C for 30 min. Cell lysates were centrifuged at 14,000 rpm in a microcentrifuge for 10 min at 4°C. The supernatants were collected, and the protein concentration was measured by Bio-Rad protein assay kit. 60 *μ*g of total proteins was processed for western blotting. Rabbit polyclonal antibodies specific for LY6E (Santa Cruz) and rabbit polyclonal antibodies for *β*-actin (Sangon Biotech) were used. Quantity analysis software (Bio-Rad Laboratories) was used for density analysis. Quantitation of LY6E protein expression was evaluated by a FluorChem FC2 system (NatureGene Corp., New Jersey, USA). Data was expressed as the ratio of LY6E integral optical density and *β*-actin integral optical density in the same sample.

### 2.6. Enzyme-Linked Immunosorbent Assay

Kidneys from mice were dissected out, chopped finely with a razor blade, and suspended in PBS. Renal cells were spun to a pellet at 1100 rpm at 4°C for 6 min. The supernatant which was enriched in interstitial fluid or “renal plasma” was stored frozen at −80°C until analysis [23]. IFN*α* protein was measured using an ELISA kit (Bairui Biotech, Shanghai, China) as per the manufacturer's protocol.

### 2.7. Histopathological Analysis

The kidneys were dissected and fixed in 10% buffered formalin, and paraffin-embedded sections of kidney tissues (3 mm thick) were stained with hematoxylin and eosin (H&E), periodic acid-Schiff (PAS), and Masson's trichrome staining for histopathological examinations. The sections were evaluated unbiasedly by an experienced pathologist, as previously described [17, 24]. The presence of glomerulonephritis was evaluated by light microscopy.

### 2.8. 24-Hour Urinary Albumin, Serum Creatinine, and Blood Urea Nitrogen Screening

Overnight urine samples were collected by metabolism cages, and albumin levels were monitored using Coomassie Brilliant Blue G-250 (Beyotime Institute of Biotechnology, Shanghai, China). The levels of serum creatinine and blood urea nitrogen were determined by colorimetric analysis kits (Gotofcm Biotech, Hangzhou, China).

### 2.9. Statistical Analysis

All data was expressed as mean ± standard deviation. A one-way analysis of variance (ANOVA) test was used for comparison of more than two groups. The differences between the groups were assessed with the Post Hoc Bonferroni test. The datasets were analyzed using the SPSS v 13.0 statistical package. Each experiment was repeated at least 3 times to assess reproducibility. A *p* value of 0.05 was considered statistically significant.

## 3. Results

### 3.1. Targeted Suppression of TWEAK Attenuated Renal Damage in MRL/lpr Mice

**Figure 1:**
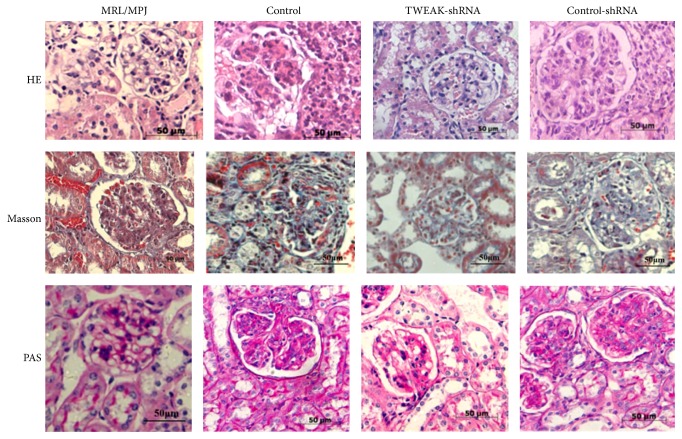
Renal histopathological changes of MRL/lpr mice were attenuated after LV-TWEAK-shRNA treatment. The kidney tissues were stained, respectively, with hematoxylin and eosin (H&E), periodic acid-Schiff (PAS), and Masson's trichrome staining for histopathological examinations. Control, MRL/lpr mice treated with normal saline; TWEAK-shRNA, MRL/lpr mice treated with LV-TWEAK-shRNA; control-shRNA, MRL/lpr mice treated with LV-control-shRNA.

We first confirmed pathological changes in the kidneys of MRL/lpr mice. As expected, compared to control MRL/MPJ mice, MRL/lpr mice showed histological evidence of severe glomerulonephritis, characterized by glomerular hypercellularity, PAS-positive material, and collagenous fiber deposition (Figure 1). We have previously showed that LV-TWEAK-shRNA treatment decreased TWEAK mRNA expression in MRL/lpr mice [25], so we investigated whether LV-TWEAK-shRNA treatment could also regulate renal damage in MRL/lpr mice. After treatment with LV-TWEAK-shRNA, we observed that glomerulonephritis was significantly improved in MRL/lpr mice, while mice that received LV-control-shRNA treatment had no alterations in the extent of renal damage (Figure 1).

We also examined the levels of 24 h urinary albumin, serum creatinine, and blood urea nitrogen among groups (Table 1). MRL/lpr mice developed higher levels of serum creatinine, blood urea nitrogen, and albuminuria than MRL/MPJ mice (*p* < 0.05). These measurements of renal damage were all reduced in MRL/lpr mice treated with LV-TWEAK-shRNA (*p* < 0.05), but not in mice treated with LV- control-shRNA. Our results suggest that TWEAK has a positive role in driving multiple pathological events associated with LN, and targeted inhibition of TWEAK expression could effectively attenuate renal damage in MRL/lpr mice.

### 3.2. TWEAK Stimulated the Activation of Type I IFN Pathway in MRL/lpr Mice

Kidneys from mice were extracted and assessed for soluble IFN*α* levels in the renal interstitial fluid or “plasma” after treatment with LV-TWEAK-shRNA. The soluble IFN*α* levels were higher in MRL/lpr mice than in MRL/MPJ mice (*p* < 0.05) (Table 2), supporting the idea that type I IFN pathway plays an important role in LN. LV-TWEAK-shRNA treatment significantly downregulated the IFN*α* levels (*p* < 0.05), while LV-control-shRNA had no effect (Table 2). Our results indicate that TWEAK could induce renal IFN*α* production in MRL/lpr mice.

**Figure 2:**
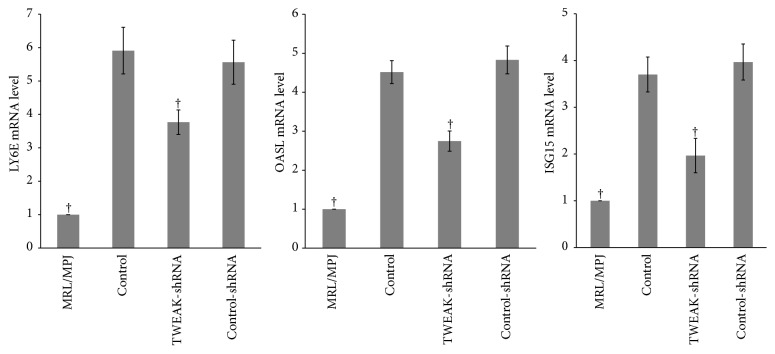
LV-TWEAK-shRNA treatment inhibited expression of type I IFN-inducible genes in MRL/lpr mice. Total RNA was extracted from kidneys and subjected to quantitative real-time PCR analysis. The ratio of target gene/*β*-actin mRNA levels in MRL/MPJ mice was designated as one. The error bars represent standard deviations. Control, MRL/lpr mice treated with normal saline; TWEAK-shRNA, MRL/lpr mice treated with LV-TWEAK-shRNA; control-shRNA, MRL/lpr mice treated with LV-control-shRNA. The numbers of MRL/MPJ, control, TWEAK-shRNA, and control-shRNA mice were 7, 10, 12, and 12, respectively. ^†^*p* < 0.05 compared with control. *p* values have been adjusted for multiplicity.

**Figure 3:**
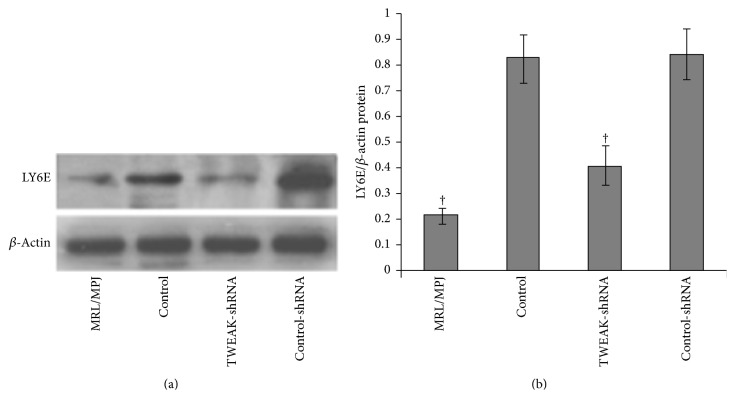
LV-TWEAK-shRNA treatment inhibited expression of LY6E protein in MRL/lpr mice. Total lysates of kidneys were collected for western blotting to measure LY6E expression. The representative experiment was showed in (a); the ratios of TWEAK/*β*-actin integral optical density were shown in (b). The error bars represent standard deviations. Control, MRL/lpr mice treated with normal saline; TWEAK-shRNA, MRL/lpr mice treated with LV-TWEAK-shRNA; control-shRNA, MRL/lpr mice treated with LV-control-shRNA. The numbers of MRL/MPJ, control, TWEAK-shRNA, and control-shRNA mice were 7, 10, 12, and 12, respectively. ^†^*p* < 0.05 compared with control. *p* values have been adjusted for multiplicity.

To confirm that TWEAK was associated with activation of type I IFN pathway in MRL/lpr mice, three type I IFN-inducible genes (LY6E, OASL, and ISG15) were examined by quantitative real-time PCR (Figure 2). Results demonstrated that renal LY6E, OASL, and ISG15 mRNA expression in MRL/lpr mice were higher than those in MRL/MPJ mice (*p* < 0.05). The expressions of LY6E, OASL, and ISG15 mRNA were downregulated after treatment with LV-TWEAK-shRNA (*p* < 0.05), but not with LV-control-shRNA. In parallel, we also examined the LY6E protein levels by western blotting of the samples derived from each group (Figure 3). MRL/lpr mice exhibited higher renal LY6E protein levels than MRL/MPJ mice (*p* < 0.05), but LV-TWEAK-shRNA treatment reduced LY6E protein expression in MRL/lpr mice (*p* < 0.05), while LV-control-shRNA treatment had no effect. These results suggest that TWEAK could activate the type I IFN pathway in MRL/lpr mice.

### 3.3. TWEAK Upregulated the Expression of Type I IFN-Inducible Gene in PBMCs Derived from LN Patients

**Figure 4:**
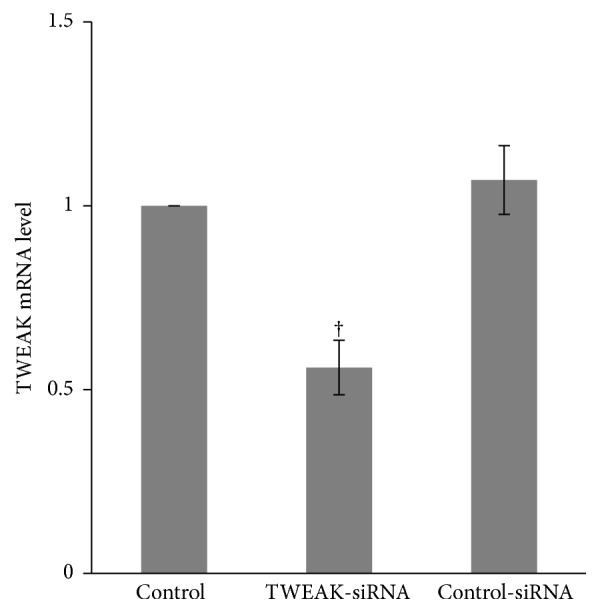
TWEAK mRNA expression was suppressed by TWEAK-siRNA-lipofectamine 2000 in PBMCs. Total RNA from PBMCs obtained from LN patients (*n* = 12) was extracted for quantitative real-time PCR. The ratio of TWEAK/*β*-actin mRNA levels was calculated. The ratio of mRNA levels in cells treated with normal saline was designated as one. The error bars represent standard deviations. Control, PBMCs treated with normal saline; TWEAK-siRNA, PBMCs treated with TWEAK-siRNA-lipofectamine 2000; control-siRNA, PBMCs treated with control-siRNA-lipofectamine 2000. ^†^*p* < 0.05 compared with control. *p* values have been adjusted for multiplicity.

**Figure 5:**
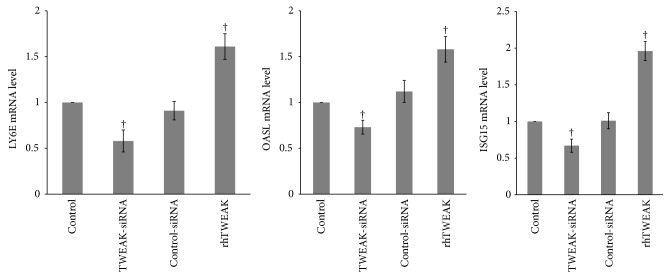
TWEAK upregulated expression of type I IFN-inducible genes in PBMCs. Total RNA from PBMCs obtained from LN patients (*n* = 12) was extracted for quantitative real-time PCR. The ratio of target gene/*β*-actin mRNA levels was calculated. The ratio of mRNA levels in cells treated with normal saline was designated as one. The error bars represent standard deviations. Control, PBMCs treated with normal saline; TWEAK-siRNA, PBMCs treated with TWEAK-siRNA-lipofectamine 2000; control-siRNA, PBMCs treated with control-siRNA-lipofectamine 2000. rhTWEAK, PBMCs treated with rhTWEAK. ^†^*p* < 0.05 compared with control. *p* values have been adjusted for multiplicity.

While we have demonstrated the effect of TWEAK on the induction of type I IFN-inducible genes in mice, we next examined this relationship in LN patient-derived PBMCs. TWEAK-siRNA-lipofectamine 2000 or control reagents were used to suppress TWEAK in PBMCs. As showed in Figure 4, TWEAK-siRNA-lipofectamine 2000 significantly decreased TWEAK mRNA level in PBMCs, but control-siRNA-lipofectamine 2000 did not. TWEAK-siRNA-lipofectamine 2000 treatment of PBMCs also significantly inhibited the expression of LY6E, OASL, and ISG15 as measured by quantitative real-time PCR (*p* < 0.05), and control-siRNA-lipofectamine 2000 treatment had no effects on any of the type I IFN-inducible genes relative to control (Figure 5). To confirm the sufficiency of TWEAK in regulating these targets, we next cultured PBMCs with rhTWEAK. Results showed that rhTWEAK treatment upregulated the expression of all three type I IFN-inducible genes (*p* < 0.05) (Figure 5). Overall, we confirmed that TWEAK could stimulate expression of type I IFN-inducible genes in patient-derived PBMCs.

## 4. Discussion

In this study, we aimed to determine if TWEAK specific inhibition or activation in preclinical models modulated activity of the type I IFN pathway in LN. Our results indicated that RNAi suppression of TWEAK expression could inhibit the activation of the type I IFN pathway in PBMCs from LN patients and in renal tissues from MRL/lpr mice. We also demonstrated that rhTWEAK stimulation was sufficient in activating this pathway as well. Given the critical roles of type I IFN signaling pathway in LN, our results provide additional rationale to clinically investigate this mechanism in LN.

Type I IFN plays a crucial role in the pathogenesis of SLE [4, 6–10, 26], and the IFN-inducible gene signature may serve as a marker for more severe disease involving the kidneys, hematopoietic cells, and the central nervous system [26]. Our research showed that levels of soluble IFN*α* and type I IFN-inducible gene expression were higher in MRL/lpr mice than those in MRL/MPJ mice, and the activation of type I IFN pathway was consistent with severe glomerulonephritis and elevated index levels of renal damage in MRL/lpr mice. Our results agree with previous reports in that type I IFN signaling may directly contribute to the pathogenesis of LN. In B6/lpr and MRL/lpr mice, sustained injection of polyinosinic : polycytidylic acid as a potent inducer of type I IFN resulted in a dramatic aggravation of the renal disease, higher titers of autoantibodies, a 10-fold increase in serum Ig, and accumulation of activated lymphocytes [9, 27]. Interferon regulatory factor 5 (IRF-5) is a member of the IRF family of transcription factors that induces the expression of type I IFN and IFN-inducible genes [28–30]. In IRF5^−/−^ MRL/lpr mice, IFN*α* and autoantibody production were markedly reduced, and glomerulonephritis was much improved [31, 32].

As a member of the TNF ligand superfamily, TWEAK mediates several important pathologic processes involved in tissue injury relating to LN [11, 33]. LN patients had significantly higher TWEAK expression in glomeruli and tubulointerstitium compared with normal controls [15]. Our results demonstrated that TWEAK inhibition attenuated renal damage in MRL/lpr mice and also suppressed the activation of type I IFN pathway. We also demonstrated this phenomenon in cultured patient-derived PBMCs cells and illustrated that TWEAK was effective and sufficient in regulating expression of type I IFN-inducible genes. In mice without prior underlying renal disease, systemic overexpression of TWEAK induced kidney inflammation and fibrosis [34]. Our results suggest that TWEAK-associated renal damage may in part require the activation of the type I IFN signaling pathway in LN. In human macrophage-like THP-1 cells, TWEAK induced the expression of inflammatory mediators, such as MMP-9, IL-6, IL-8, and monocyte chemotactic protein 1 (MCP-1) [35]. These activities could confer the ability of TWEAK to induce renal cells and immunocytes in secreting proinflammatory chemokines and cytokines, leading to renal damage in SLE.

In the chronic graft-versus-host model of SLE, mice treated with an anti-TWEAK neutralizing mAb had significantly downregulated kidney expression of MCP-1, IL-4, IL-6, and proteinuria, as well as glomerular IgG deposition [36]. TWEAK acts through its receptor fibroblast growth factor-inducible 14 (Fn14). After Fn14 was knockout, female MRL/lpr mice showed reduced levels of proteinuria, decreasing glomerular Ig deposition, and alleviated renal histopathology accompanied by attenuated glomerular and tubulointerstitial inflammation [37]. In our study, downregulating expression of TWEAK gene in MRL/lpr mice significantly decreased the levels of serum creatinine, blood urea nitrogen, and albuminuria and improved kidney histology, including a substantial reduction in infiltrated inflammatory cell, PAS-positive material, and collagenous fiber deposition. Overall, these evidences indicate inhibiting the TWEAK/Fn14 axis represents a viable therapeutic strategy in treating LN patients.

## 5. Conclusion

In conclusion, our results showed that TWEAK regulated renal damage, and this was associated with activation of the type I IFN pathway. This provides preclinical evidence to further examine TWEAK in SLE. Additionally, our implementation of genetic targeting TWEAK represents a novel therapeutic intervention in LN. The detailed mechanisms in which TWEAK regulates the type I IFN pathway should be characterized and may identify parallel approaches to target TWEAK in patients.

## Figures and Tables

**Table 1:** The index levels of renal damage in different groups of MRL/lpr mice.

	24-hour urinary albumin (mg)	Scr (mmol/L)	BUN (*μ*mol/L)
MRL/MPJ	1.45 ± 0.34^†^	5.52 ± 0.81^†^	42.36 ± 5.32^†^
Control	8.20 ± 1.12	9.845 ± 1.33	103.96 ± 12.18
TWEAK-shRNA	3.86 ± 0.77^†^	7.13 ± 0.91^†^	63.74 ± 5.04^†^
Control-shRNA	8.50 ± 1.32	10.06 ± 1.21	95.93 ± 11.52

Data is presented as mean ± standard deviation. Control, MRL/lpr mice treated with normal saline; TWEAK-shRNA, MRL/lpr mice treated with LV-TWEAK-shRNA; control-shRNA, MRL/lpr mice treated with LV-control-shRNA. The numbers of MRL/MPJ, control, TWEAK-shRNA, and control-shRNA mice were 7, 10, 12, and 12, respectively. ^†^*p* < 0.05 compared with control. *p* values have been adjusted for multiplicity.

**Table 2:** Effects of LV-TWEAK-shRNA treatment on soluble IFN*α* levels in MRL/lpr mice.

Groups	Soluble IFN*α* levels (pg/ml)
MRL/MPJ	27.62 ± 2.53^†^
Control	52.61 ± 3.72
TWEAK-shRNA	35.64 ± 1.46^†^
Control-shRNA	51.46 ± 6.76

Data is presented as mean ± standard deviation. Control, MRL/lpr mice treated with normal saline; TWEAK-shRNA, MRL/lpr mice treated with LV-TWEAK-shRNA; control-shRNA, MRL/lpr mice treated with LV-control-shRNA. The numbers of MRL/MPJ, control, TWEAK-shRNA, and control-shRNA mice were 7, 10, 12, and 12, respectively. ^†^*p* < 0.05 compared with control. *p* values have been adjusted for multiplicity.

## Abbreviations

Ct: Cycle threshold
H&E: Hematoxylin and eosin
IFN: Interferon
IL: Interleukin
IRF-5: Interferon regulatory factor 5
ISG15: IFN*α*-inducible protein (clone IFI-15K)
LN: Lupus nephritis
LY6E: Lymphocyte antigen 6 complex locus E
MCP-1: Monocyte chemotactic protein 1
TWEAK: Tumor necrosis factor-like weak inducer of apoptosis
OASL: 2′,5′-Oligoadenylate synthetase-like
PAS: Periodic acid-Schiff
PBMCs: Peripheral blood mononuclear cells
rhTWEAK: Recombinant human TWEAK
SLE: Systemic lupus erythematosus
TLR: Toll-like receptors.

## Supplementary Materials

Supplementary Materials Supplementary Table  1. Demographic data of study population. Supplementary Table  2. The sequences of shRNA. Supplementary Table  3. The sequences of primers.  
