# Supplementary material for: Tumor Necrosis Factor-Like Weak Inducer of Apoptosis Activates Type I Interferon Signals in Lupus Nephritis
Source: Biomed Res Int. 2017 Nov 26;2017:4927376. doi: 10.1155/2017/4927376 (PMC5733219; doi:10.1155/2017/4927376)
Supplement: Supplementary Materials — Supplementary Table 1. Demographic data of study population. Supplementary Table 2. The sequences of shRNA. Supplementary Table 3. The sequences of primers. [file 4927376.f1.doc]

**Supplementary Table 1. Demographic data of Study population.**

| Patient  number | Gender | Age (years) | Ethnicity | Symptoms duration until diagnosed | 24-hour urinary albumin (grams) | Renal pathological type | SLEDAI  2000 |
| --- | --- | --- | --- | --- | --- | --- | --- |
| 1 | Female | 43 | Han | 20 months | 3.31 | V | 10 |
| 2 | Female | 39 | Han | 8 days | 1.18 | V | 9 |
| 3 | Female | 46 | Han | 1 month | 7.86 | III | 14 |
| 4 | Female | 40 | Han | 3 months | 7.11 | IV | 12 |
| 5 | Female | 39 | Han | 3 months | 11.75 | --^a^ | 18 |
| 6 | Female | 30 | Han | 2 months | 1.16 | -- | 20 |
| 7 | Female | 14 | Han | 4 months | 1.68 | -- | 19 |
| 8 | Female | 42 | Han | 2 months | 4.9 | -- | 12 |
| 9 | Female | 25 | Han | 1 month | 1.04 | IV | 20 |
| 10^b^ | Female | 38 | Han | 36 months | 1.14 | -- | 8 |
| 11 | Female | 38 | Han | 1 month | 7.52 | IV | 12 |
| 12 | Female | 48 | Han | 1 month | 5.96 | IV & V | 11 |

^a^ the patient refused percutaneous renal biopsy by B-mode ultrasonography guiding.

^b^ the patient originally presented with purpura, and was diagnosed immune related thrombocytopenic purpura and prescribed prednisone alone; the treatment was effective, and discontinued after 24 months. But after 10 months, the patient again presented with purpura acompanied with albuminuria.

**Supplementary Table 2. The sequences of shRNA**

| Gene | Sequence |
| --- | --- |
| LV-TWEAK-shRNA | Forward: 5′-CGG TAA CCT ACT TTG GAC TCT TTC CTC GAG GAA AGA GTC CAA AGT AGG TTA TTT TTG-3′ |
|  | Reverse: 5′-AAT TCA AAA AAA TAC TTT GGA CTC TTT CCT CGA GGA AAG AGT CCA AAG TAG GTT A-3′ |
| LV-control-shRNA | Forward: 5′-CCG GTT CTC CGA ACG TGT CAC GTT TCA AGA GAA CGT GAC ACG TTC GGA GAA TTT TTG-3ˊ |
|  | Reverse: 5′-AAT TCA AAA ATT CTC CGA ACG TGT CAC GTT CTC TTG AAA CGT GAC ACG TTC GGA GAA-3′ |

**Supplementary Table 3. The sequences of primers**

| Gene | Sequence | Length |
| --- | --- | --- |
| Mouse LY6E | Forward: 5′-GCT CCT CCT GGG TGT GTC TA-3′ | 117 bps |
|  | Reverse: 5′-GGC TCT GGA AGC AAG GGT A-3′ |  |
| Mouse OASL | Forward: 5′-GCG GTA GGC ATC AGA GAA AT-3′ | 164 bps |
|  | Reverse: 5′-TGA GGG TCA CAA CAT CCA CA-3′ |  |
| Mouse ISG15 | Forward: 5′-CAG ATT GCC CAG AAG ATT GG-3′ | 175 bps |
|  | Reverse: 5′-CCC TTT CGT TCC TCA CCA G-3′ |  |
| Mouse β-actin | Forward: 5′-GAG ACC TTC AAC ACC CCA GC-3′ | 263 bps |
|  | Reverse: 5′-ATG TCA CGC ACG ATT TCC C-3′ |  |
| Human TWEAK | Forward: 5′-GAG GAA GCC AGA TCA ACA G-3′ | 157 bps |
|  | Reverse: 5′-CAC CAT CCA CCA GCA AGT C-3′ |  |
| Human LY6E | Forward: 5′-CCA GGA CAA CTA CTG CGT GAC-3′ | 135 bps |
|  | Reverse: 5′-ATG GAA GCC ACA CCA ACA TT-3′ |  |
| Human OASL | Forward: 5′- GAC TCC TTC GTG GCT CAG TG-3′ | 103 bps |
|  | Reverse: 5′- GGA AAT GCT CCT GCC TCA G -3′ |  |
| Human ISG15 | Forward: 5′-AAA TGC GAC GAA CCT CTG-3′ | 175 bps |
|  | Reverse: 5′-CGC TCA CTT GCT GCT TCA-3′ |  |
| Human β-actin | Forward: 5′-TGA CGT GGA CAT CCG CAA AG-3′ | 205 bps |
|  | Reverse: 5′-CTG GAA GGT GGA CAG CGA GG-3′ |  |
